# Weighted gene co-expression network analysis revealed key biomarkers associated with the diagnosis of hypertrophic cardiomyopathy

**DOI:** 10.1186/s41065-020-00155-9

**Published:** 2020-10-24

**Authors:** Xin Li, Chenxin Wang, Xiaoqing Zhang, Jiali Liu, Yu Wang, Chunpu Li, Dongmei Guo

**Affiliations:** 1 Department of Cardiovascular, The Third Central Hospital of Tianjin, Tianjin, China; 2 Department of Respiratory medicine, The Third Central Hospital of Tianjin, Tianjin, China; 3 Department of internal medicine, Affiliated Hospital of Nankai University, Tianjin, China; 4 Department of Hematology, Taian City Central Hospital, 29 Longtan Road, Taian, 271000 Shandong China; 5 Department of Orthopedics, Taian City Central Hospital, 29 Longtan Road, Taian, 271000 Shandong China

**Keywords:** Hypertrophic cardiomyopathy, WGCNA, Hub gene

## Abstract

**Objective:**

To reveal the molecular mechanism underlying the pathogenesis of HCM and find new effective therapeutic strategies using a systematic biological approach.

**Methods:**

The WGCNA algorithm was applied to building the co-expression network of HCM samples. A sample cluster analysis was performed using the hclust tool and a co-expression module was constructed. The WGCNA algorithm was used to study the interactive connection between co-expression modules and draw a heat map to show the strength of interactions between modules. The genetic information of the respective modules was mapped to the associated GO terms and KEGG pathways, and the Hub Genes with the highest connectivity in each module were identified. The Wilcoxon test was used to verify the expression level of hub genes between HCM and normal samples, and the “pROC” R package was used to verify the possibility of hub genes as biomarkers. Finally, the potential functions of hub genes were analyzed by GSEA software.

**Results:**

Seven co-expression modules were constructed using sample clustering analysis. GO and KEGG enrichment analysis judged that the turquoise module is an important module. The hub genes of each module are RPL35A for module Black, FH for module Blue, PREI3 for module Brown, CREB1 for module Green, LOC641848 for module Pink, MYH7 for module Turquoise and MYL6 for module Yellow. The results of the differential expression analysis indicate that MYH7 and FH are considered true hub genes. In addition, the ROC curves revealed their high diagnostic value as biomarkers for HCM. Finally, in the results of the GSEA analysis, MYH7 and FH highly expressed genes were enriched with the “proteasome” and a “PPAR signaling pathway,” respectively.

**Conclusions:**

The MYH7 and FH genes may be the true hub genes of HCM. Their respective enriched pathways, namely the “proteasome” and the “PPAR signaling pathway,” may play an important role in the development of HCM.

## Introduction

Hypertrophic cardiomyopathy (HCM) is the most common genetic heart disease with a prevalence of approximately 1:500 [18]. HCM is characterized by ventricular hypertrophy, with clinical features in patients ranging from asymptomatic to heart failure and sudden cardiac death. At present, symptomatic treatment is still used to delay the progress of the disease, while traditional treatment is still unable to reverse the disease. Since the first chromosomal location was mapped in 1989 [13], variants in numerous genes have been reported to cause HCM. The clinical diagnostic for genetic testing of HCM is increasingly becoming a part of the mainstream clinical management of patients and playing a key role in the cascade detection of family members [5, 10]. Previous studies have shown that in more than 50% of HCM samples, the disease is caused by mutations in genes encoding cardiac myosin, such as MYH7, TNNI3, MYBPC3 and MYL3. However, the molecular mechanism underlying the pathogenesis of HCM remains unclear, and new advanced analytic strategies and genomic technologies provide opportunities to explore the genetic structure of HCM. Therefore, this is an opportunity to reveal the molecular mechanism of HCM and find new effective therapeutic strategies.

In a number of computational studies, disease risk modules have been developed to provide important measurement for the diagnosis of genetic mutations and the development of new treatment strategies [11, 27, 28]. WGCNA (weighted gene co-expression network analysis) is a powerful genetic analysis strategy. It is a systematic biological analysis method based on “guilt-by-association.” This method is used to identify gene modules that can be used as candidate biomarkers or therapeutic targets [8, 16]. It is currently widely used for research and analysis of schizophrenia [7, 21], cancer [6], intracranial aneurysm [29] and other diseases. By using WGCNA, we can create co-expression networks to find differentially relevant gene clusters and carry out gene-specific analysis [2, 27, 28].

In this study, we used this method to analyze a large amount of HCM genetic data to find genes and pathways that play an important role in the occurrence and development of HCM. Provide guidance for disease research and identify potential effective treatment options. It is hoped that the results of this study will provide guidance for the study of the disease and the search for potential effective treatments.

## Materials and methods

### Microarray data analysis

Analysis was carried out of the gene expressions of the HCM datasets acquired from the GEO database (http://www.ncbi.nlm.nih.gov/geo )[3]. GSE36961, a much larger and newer microarray dataset of HCM, includes 106 cases and 39 controls (https://www.ncbi.nlm.nih.gov/geo/query/acc.cgi?acc=GSE36961). Clinical information, including age and gender, was also acquired. The data set was based on Platforms GPL15389 (Illumina humanHT-12 V3.0 expression beadchip). Gene IDs were mapped to the microarray probes using the annotated information offered by the record. Probes corresponding to more than one gene were excluded from the dataset. The average expression values of the genes were obtained using measurements from a number of probes. A suitable threshold value was selected based on the number of probes with different thresholds of expression. The WGCNA algorithm [16] was applied to building the co-expression network of HCM samples. Sample cluster analysis was performed using the hclust tool (R package, https://www.rdocumentation.org/packages/stats/versions/3.6.1/topics/hclust) with a threshold value of 35.

### Co-expression module construction

The power value was evaluated during the construction of the modules using the WGCNA package in R (https://cran.r-project.org/web/packages/WGCNA/). The mean connectivity and scale independence of network modules were analyzed using the gradient test under different power values, which ranged from 1 to 20. The soft threshold power of 9 was chosen based on the scale-free topology criterion. The WGCNA algorithm further identified co-expression modules under these conditions. The minimum size of the gene group was set at 50 to ensure the reliability of the results for this module.

### Interaction analysis of co-expression modules

The interactive connection among the co-expression modules was studied using the WGCNA algorithm. The WGCNA R software package [16] can be used to determine network construction, the calculation of topological properties, gene selection, module detection, differential network analysis, and network statistics. Furthermore, a heat map was plotted to exhibit the strength of interaction among the modules.

### Functional enrichment analysis

Functional enrichment analysis was carried out in co-expression modules. The genetic information of the respective modules was mapped to the associated gene ontology (GO) terms and KEGG pathways using the DAVID tool (version 6.8; http://david.abcc.ncifcrf.gov/) [12]. The top five records with *p*-value < 0.05 were retained for analysis.

### Hub gene identification and validation

Hub Genes with the highest connectivity in each module were identified using the function “chooseTopHubInEachModule” in WGCNA [23]. Bee swarm plots were created using the R package (https://cran.r-project.org/web/packages/beeswarm/index.html). Wilcoxon tests were performed to validate the hub genes’ expression levels between HCM and normal tissue samples. *P* < 0.05 was considered statistically significant. To validate the possibility of a hub gene as a biomarker, we outlined receiver operating characteristic (ROC) curves and calculated the area under the ROC curve (AUC) with the “pROC” R package [24].

### Gene set enrichment analysis

To detect potential function of the hub genes, gene set enrichment analysis (GSEA) was conducted to explore the high-risk score associated KEGG pathways on GSEA software downloaded from the Broad Institute (http://www.broadinstitute. org/gsea). By running GSEA, normalized enrichment scores and *p*-value were generated.

## Results

### HCM dataset pre-processing

**Fig. 1:**
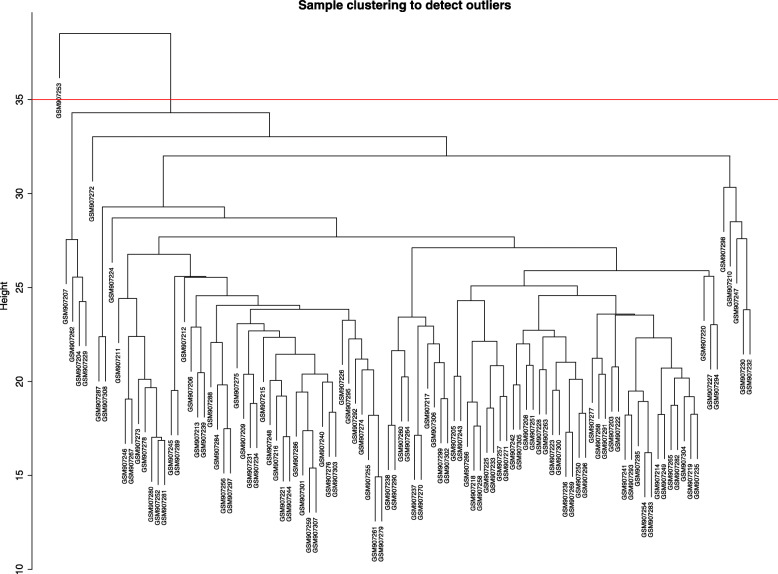
Hierarchical clustering of the top 4000 genes with the highest average expression values of HCM samples in the clustering analysis. There were 1 outlier samples in the total 106 samples, that is GSM907253, when the threshold value was determined as 35 (red line)

A total amount of 37,846 gene expression values were derived from the raw file.Four thousand genes with the greatest average expression values were chosen for cluster evaluation (Fig. 1). To ensure that the results of network construction were reliable, 105 HCM samples remained for subsequent WGCNA analysis after GSM 907253 (which was distant from other samples in the sample cluster analysis) was removed.

### Identification of co-expression modules of HCM genes

**Fig. 2:**
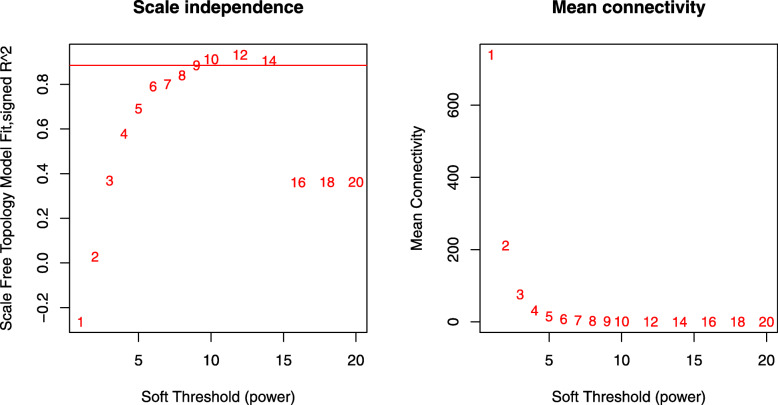
The influence of different soft threshold power on the scale independence degree of coexpression modules of HCM genes. The influence of different soft threshold power on the average connectivity degree of coexpression modules of HCM genes

**Fig. 3:**
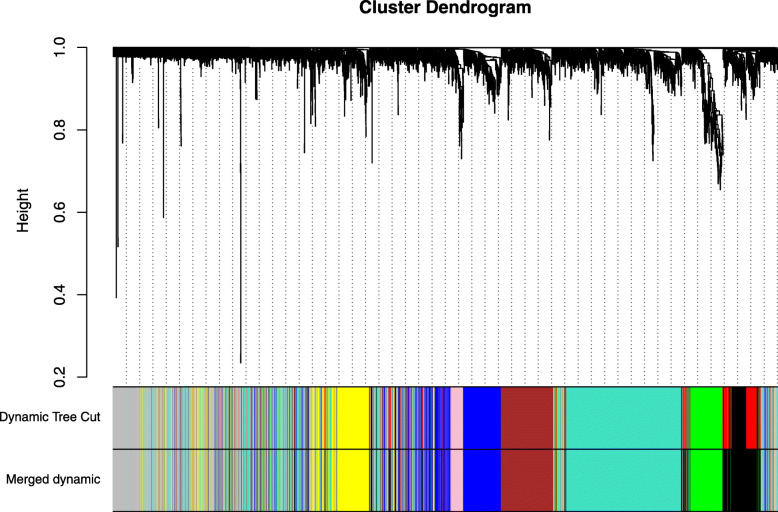
The constructed coexpression modules of HCM genes by WGCNA software

The expression values of 4000 genes in 105 HCM samples were analyzed to identify the modules of highly correlated genes. The soft threshold power was set at 9 (scale-free R2 = 0.88) to guarantee a scale-free network (Fig. 2). A total of 8 modules including green (255 genes), turquoise (992 genes), grey (932 genes), blue (473 genes), brown (424 genes), yellow (387 genes), pink (149 genes), and black (388 genes) were identified (Fig. 3). The genes in grey were not included in any module, so no further analysis was conducted for these genes.

### Correlation analysis of co-expression modules

**Fig. 4:**
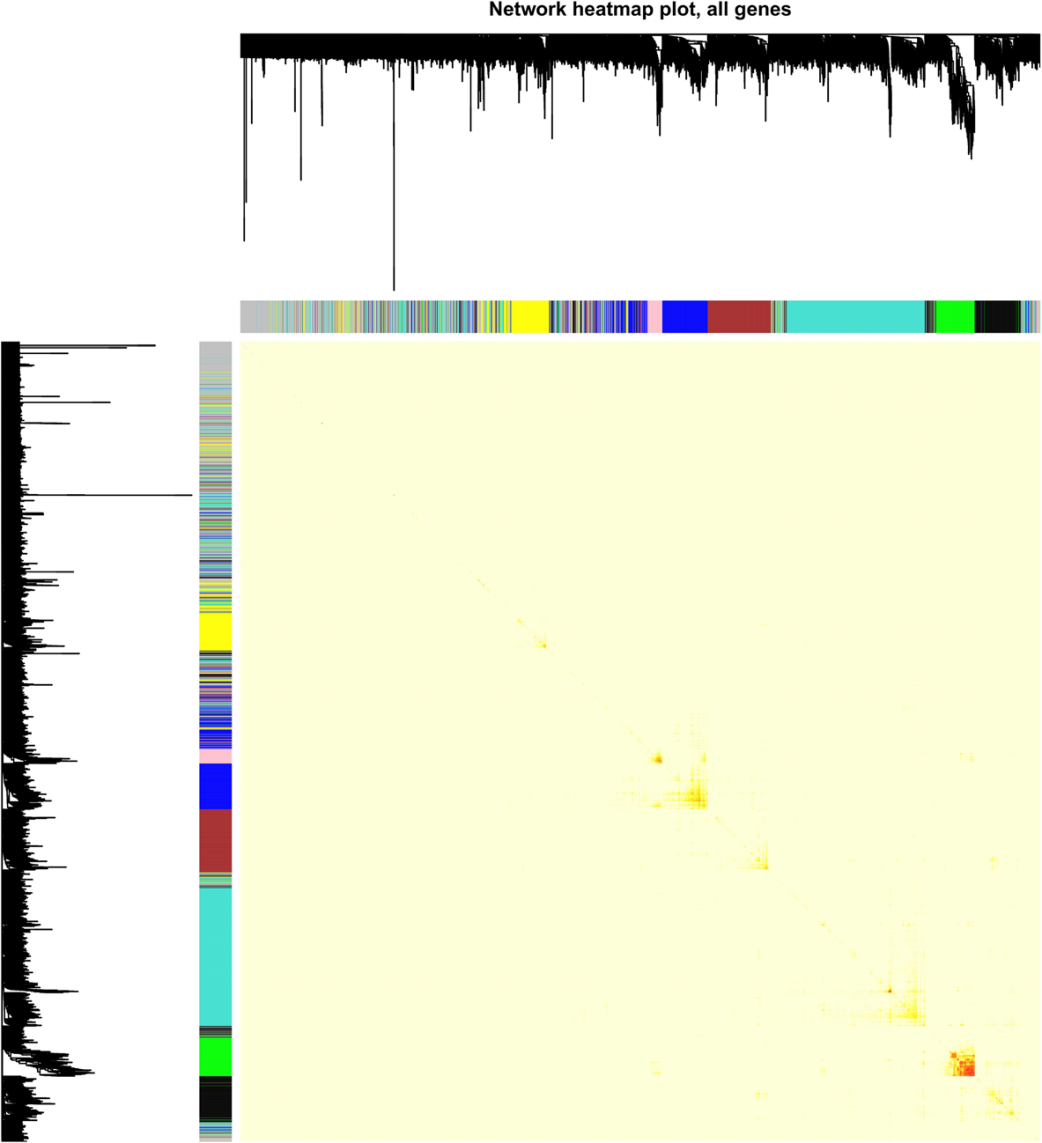
Interaction analysis between gene coexpression modules. The heatmap showed the Topological Overlap Matrix (TOM) among genes in the analysis. Different colors on the x-axis and y-axis represented different modules. The yellow brightness of the middle part represented the strength of connections between modules

**Fig. 5:**
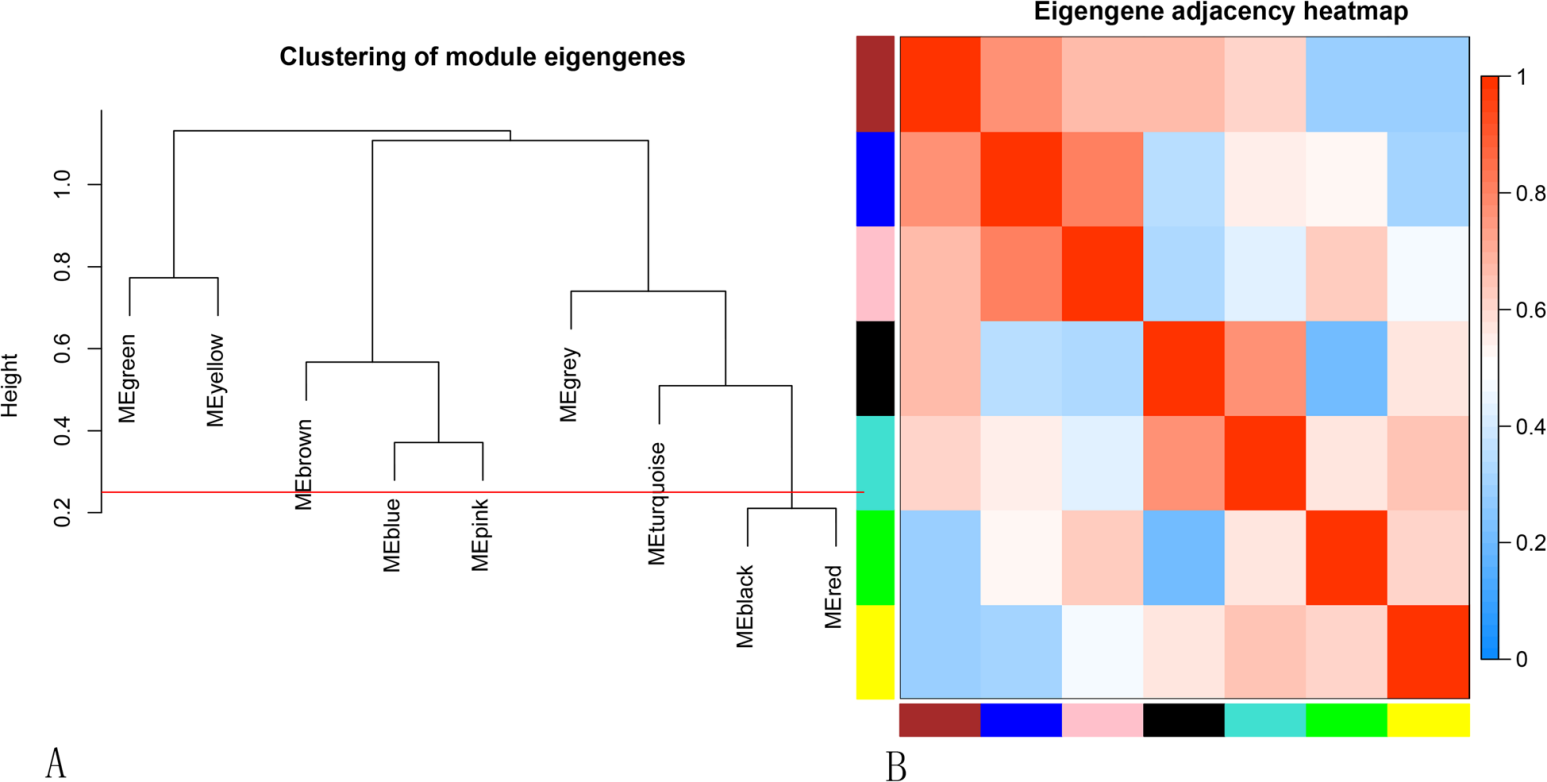
Connectivity analysis between different modules. **a** Hierarchical cluster analysis of the genes in different modules; **b** Connectivity level analysis of the genes in different modules. Within the heatmap, red represents a positive correlation and blue represents a negative correlation. Squares of red color along the diagonal are the meta-modules

The WGCNA package analyzed the interactive relationships underlying the seven co-expression modules (Fig. 4). Gene expression levels were relatively independent as illustrated by the topological overlap matrix (TOM) plot of 4000 genes, suggesting that each module was independently validated. The connectivity degree of eigengenes was assessed to further quantify the similarity of co-expression. These seven modules yielded two main clusters followed by cluster analysis (Fig. 5a), including two modules (modules Green and Yellow) and five modules (modules Brown, Blue, Pink, Turquoise and Black), respectively. Based on the heatmap plot of the adjacencies (Fig. 5b), we found two pairs (modules Blue and Pink; modules Black and Turquoise) had the higher adjacency value.

### Functional enrichment analysis in critical co-expression modules

**Fig. 6:**
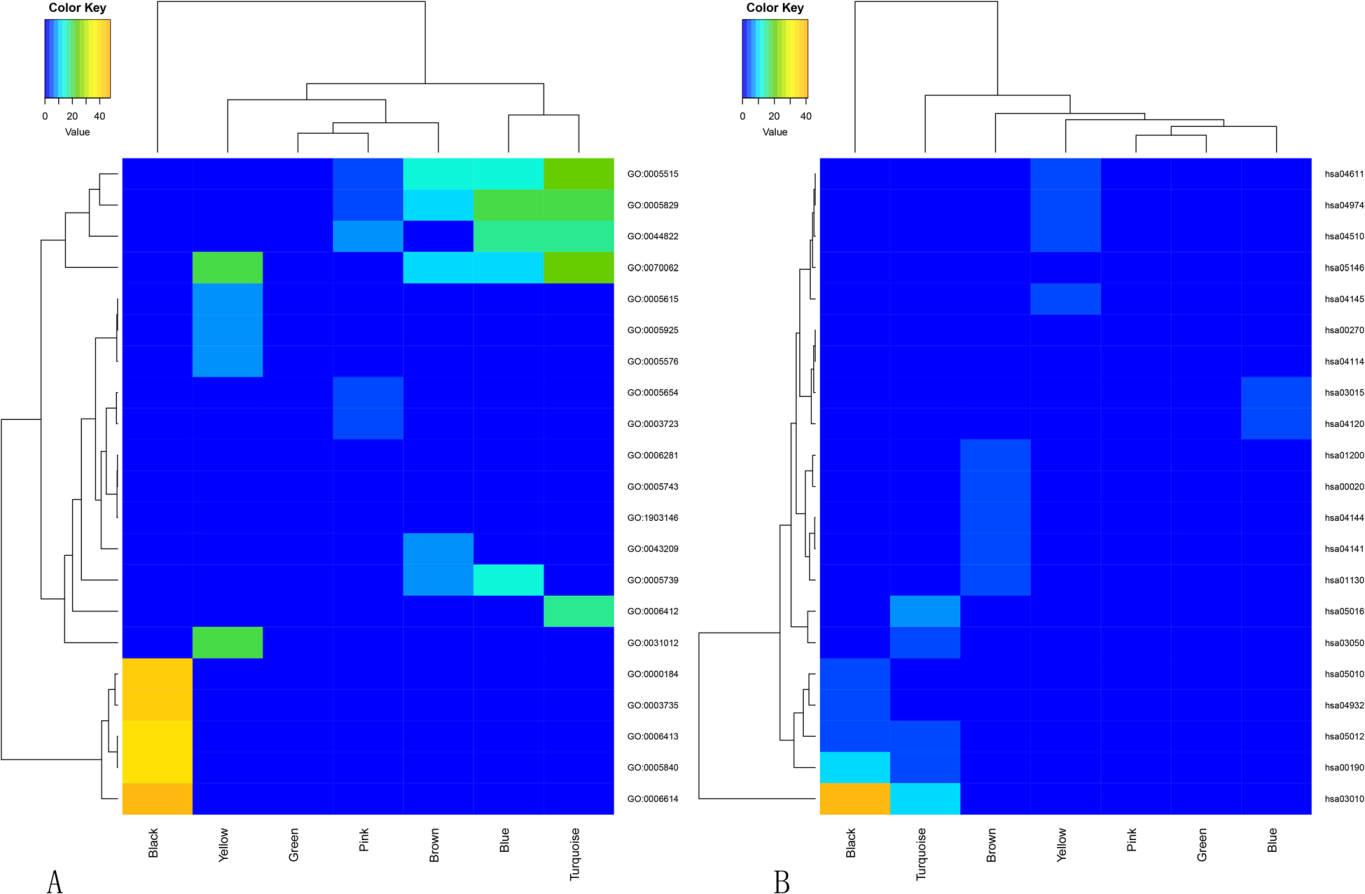
The heatmap for GO (**a**) and KEGG (**b**) enrichment analysis of HCM genes in coexpression modules. Rows and columns represent the terms and modules, respectively

Enrichment analysis of GO and KEGG were conducted to assess the functions of genes in the seven identified modules. The leading five enriched GO and KEGG terms with *p* value < 0.05 were selected for further analysis. Based on the heatmap plot for GO (Fig. 6a) and KEGG (Fig. 6b) evaluations, significant differences in the enriched terms and enriched degree was detected among the co-expression modules. Through the analysis of the GO biological process, each module was very different from the others (Table 1). The genes in module Black were generally enriched in GO:0006614 (SRP-dependent co-translational protein targeting membranes), GO:0000184 (nuclear-transcribed mRNA catabolic process, nonsense-mediated decay) and GO:0006413 (translational initiation). The genes in module Green were primarily enriched in GO:0006281 (DNA repair) and GO:1903146 (regulation of mitophagy). The genes in module Turquoise were mainly enriched in GO:0006412 (translation). The genes in the other four modules were mainly enriched in GO cellular component and GO molecular function. The results of the KEGG pathway enrichment analysis are shown in Table 2. Module Black and Turquoise were mainly enriched in the pathways hsa03010: Ribosome. Module Blue was mainly enriched in the pathways hsa04120: Ubiquitin mediated proteolysis. Module Brown was mostly enriched in cellular processes hsa01200: Carbon metabolism. Module Green was enriched in pathways hsa00190: Oxidative phosphorylation. Module Pink was mainly enriched in hsa00020: Citrate cycle (TCA cycle). In module Yellow, a total of 14 genes were mainly enriched in hsa04145: Phagosome.

**Table 1:** GO enrichment for the genes in the coexpression modules of HCM

	Term	Count	Percentage%	*p* value
Module black	GO:0006614 ~ SRP-dependent cotranslational protein targeting to membrane	42	13.72549	2.17E-48
	GO:0000184 ~ nuclear-transcribed mRNA catabolic process, nonsense-mediated decay	43	14.05229	7.92E-45
	GO:0003735 ~ structural constituent of ribosome	52	16.99345	1.92E-43
	GO:0006413 ~ translation initiation	42	13.72549	2.32E-40
	GO:0005840 ~ ribosome	44	14.37908	3.65E-40
Module blue	GO:0005829 ~ cytosol	154	38.11881	7.31E-23
	GO:0044822 ~ poly(A) RNA binding	75	18.56436	3.88E-17
	GO:0005515 ~ protein binding	273	67.57426	8.90E-15
	GO:0005739 ~ mitochondrion	75	18.56436	2.61E-14
	GO:0070062 ~ extracellular exosome	115	28.46535	3.13E-12
Module brown	GO:0005515 ~ protein binding	239	67.12483	4.32E-14
	GO:0070062 ~ extracellular exosome	102	28.65169	1.69E-11
	GO:0005829 ~ cytosol	112	31.46067	9.05E-11
	GO:0005739 ~ mitochondrion	58	16.29213	3.13E-09
	GO:0043209 ~ myelin sheath	16	4.494382	1.75E-07
Module green	GO:0006281 ~ DNA repair	9	4.945055	0.001606
	GO:0005739 ~ mitochondrion	24	13.18681	0.001903
	GO:0005743 ~ mitochondrial inner membrance	12	6.593407	0.002201
	GO:1903146 ~ regulation of mitophagy	4	2.197802	0.003498
	GO:0005515 ~ protein binding	101	55.49451	0.003597
Module pink	GO:0044822 ~ poly(A) RNA binding	24	24	1.27E-08
	GO:0005515 ~ protein binding	70	70	5.93E-07
	GO:0005829 ~ cytosol	36	36	4.25E-06
	GO:0005654 ~ nucleoplasm	32	32	6.74E-06
	GO:0003723 ~ RNA binding	13	13	2.91E-05
Module turquoise	GO:0070062 ~ extracellular exosome	237	28.31541	1.66E-24
	GO:0005515 ~ protein binding	535	63.91876	6.94E-24
	GO:0005829 ~ cytosol	259	30.94385	3.09E-22
	GO:0044822 ~ poly(A) RNA binding	122	14.57587	2.18E-19
	GO:0006412 ~ translation	49	5.854241	2.69E-17
Module yellow	GO:0070062 ~ extracellular exosome	125	36.54971	6.04E-23
	GO:0031012 ~ extracellular matrix	40	11.69591	2.10E-22
	GO:0005615 ~ extracellular space	59	17.25146	7.14E-10
	GO:0005925 ~ focal adhesion	29	8.479532	8.16E-10
	GO:0005576 ~ extracellular region	65	19.00585	2.04E-09

*Abbreviations: GO* Gene Ontology, *SRP* signal recognition particle

**Table 2:** KEGG pathway enrichment for the genes in the coexpression modules of HCM

	Term	Count	Percentage%	*p* value
Module black	hsa03010:Ribosome	48	15.68627	7.55E-42
	hsa00190:Oxidative phosphorylation	22	7.189542	1.93E-11
	hsa05010: Alzheimer’s disease	17	5.555556	6.60E-06
	hsa05012: Parkinson’s disease	15	4.901961	1.72E-05
	hsa04932: Non-alcoholic fatty liver disease (NAFLD)	15	4.901961	3.45E-05
Module blue	hsa04120: Ubiquitin mediated proteolysis	14	3.465347	1.61E-04
	hsa03015: mRNA surveillance pathway	10	2.475248	0.001237
	hsa00270: Cysteine and methionine metabolism	6	1.485149	0.004544
	hsa04114: Oocyte meiosis	10	2.475248	0.004845
	hsa01130: Biosynthesis of antibiotics	14	3.465347	0.008644
Module brown	hsa01200: Carbon metabolism	13	3.651685	4.17E-06
	hsa00020: Citrate cycle (TCA cycle)	7	1.966292	3.22E-05
	hsa04144: Endocytosis	15	4.213483	5.62E-04
	hsa04141: Protein processing in endoplasmic reticulum	12	3.370787	8.83E-04
	hsa01130: Biosynthesis of antibiotics	13	3.651685	0.001751
Module green	hsa00190: Oxidative phosphorylation	7	3.846154	0.001913
	hsa05010: Alzheimer’s disease	7	3.846154	0.00608
	hsa05016: Huntington’s disease	7	3.846154	0.011398
	hsa05012: Parkinson’s disease	6	3.296703	0.012855
Module pink	hsa00020: Citrate cycle (TCA cycle)	3	3	0.015527
	hsa01200: Carbon metabolism	4	4	0.035113
Module turquoise	hsa03010: Ribosome	31	3.703704	4.60E-11
	hsa05016: Huntington’s disease	31	3.703704	2.27E-07
	hsa03050: Proteasome	12	1.433692	2.10E-05
	hsa05012: Parkinson’s disease	21	2.508961	1.12E-04
	hsa00190: Oxidative phosphorylation	20	2.389486	1.37E-04
Module yellow	hsa04145: Phagosome	14	4.093567	5.39E-05
	hsa04510: Focal adhesion	15	4.385965	3.75E-04
	hsa04611: Platelet activation	11	3.216374	0.001026
	hsa04974: Protein digestion and absorption	9	2.631579	0.00112
	hsa05146: Amoebiasis	9	2.631579	0.003661

*Abbreviations*: *KEGG* Kyoto Encyclopedia of Genes and Genomes

### Hub gene identification and validation

**Fig. 7:**
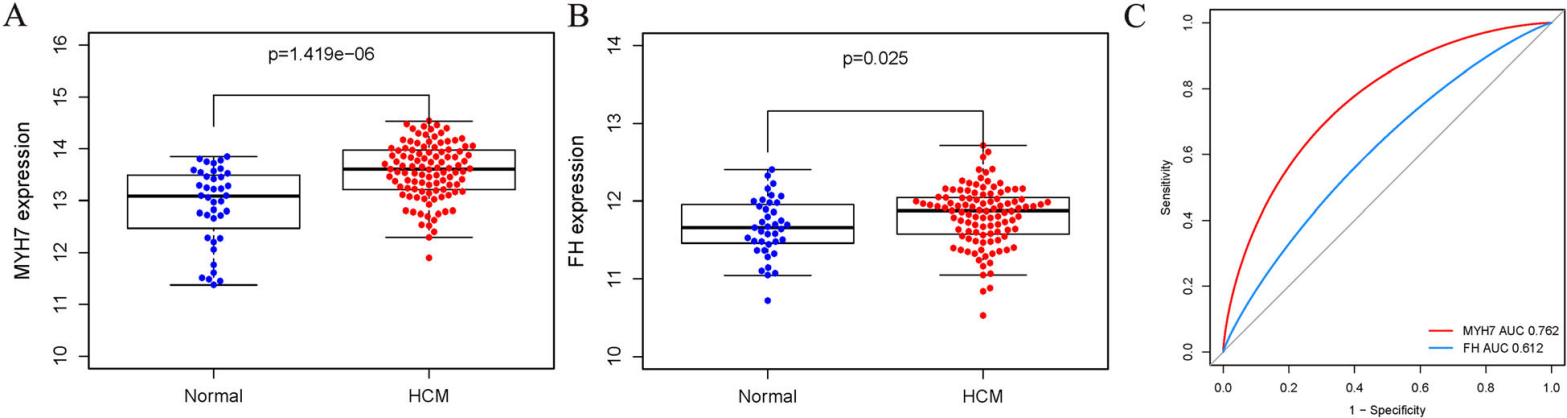
Differential expression analysis of real hub genes between HCM and normal tissues. Expression levels of MYH7 (**a**) and FH (**b**) were significantly up-regulated in HCM samples in comparison to normal tissues in the GSE36961. **c** ROC analysis of real hub genes

Modular hub genes with the highest connectivity were identified using the WGCNA package function “chooseTopHubInEachModule”. The hub genes of each module are RPL35A for module Black, FH for module Blue, PREI3 for module Brown, CREB1 for module Green, LOC641848 for module Pink, MYH7 for module Turquoise and MYL6 for module Yellow. Differential expression analysis between HCM and normal tissue samples of these hub genes was performed to evaluate their effects. Briefly, only two hub genes showed significantly higher expression compared to the normal group (*p* < 0.05). MYH7 (Fig. 7a) and FH (Fig. 7b) were regarded as true hub genes. Moreover, ROC curves revealed their high diagnostic value as biomarkers for HCM (Fig. 7c; MYH7 AUC: 0.762, FH AUC: 0.612).

### Gene set enrichment analysis

**Fig. 8:**
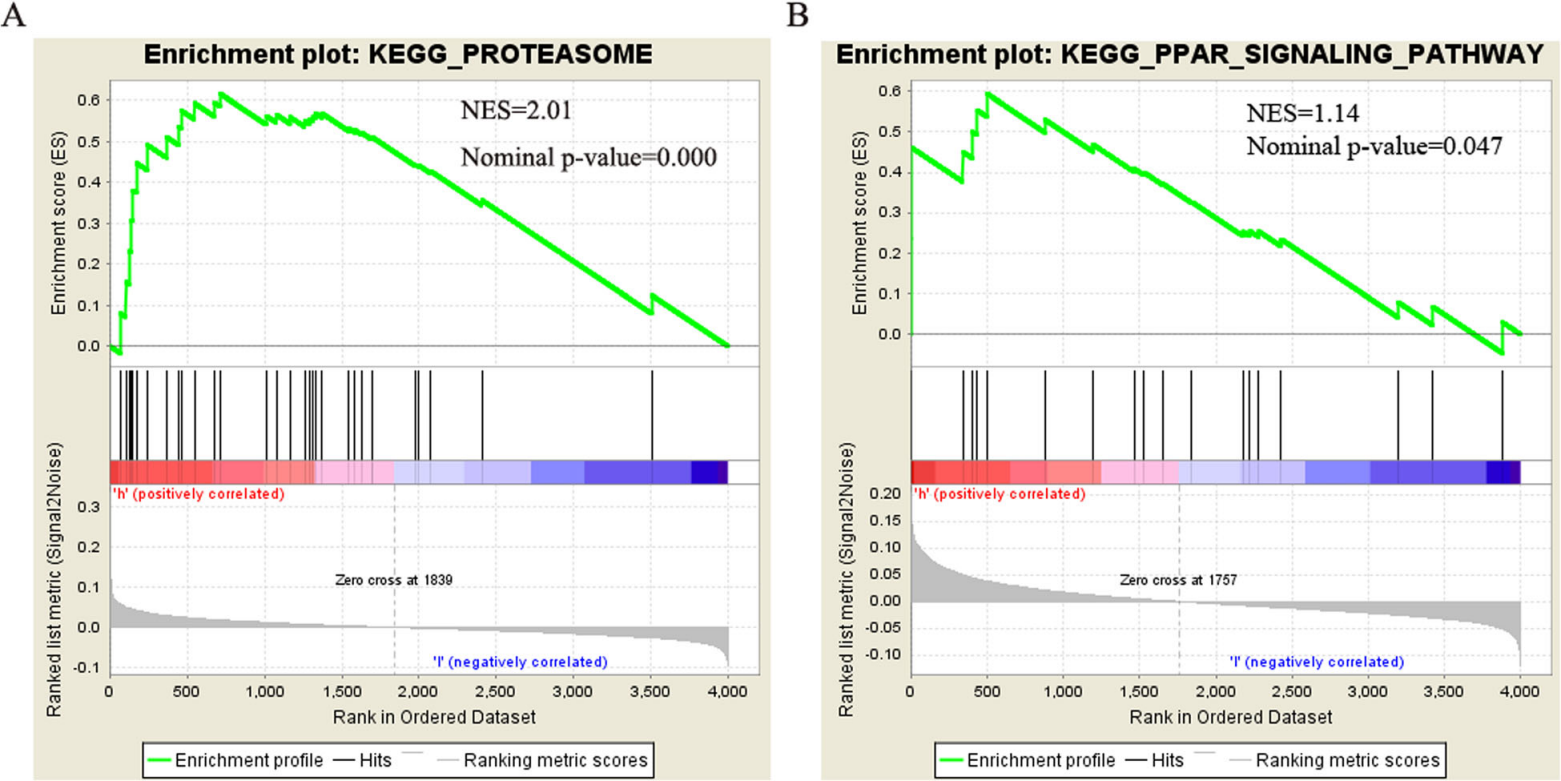
The GSEA analysis results on real hub genes. **a** High MYH7 expression was associated with Proteasome pathway. **b** High FH expression was associated with PPAR signaling pathway

We performed GSEA to further explore the potential functions of MYH7 and FH in HCM. As shown in Fig. 8a and b, genes in high expression groups of MYH7 and FH were enriched (p < 0.05) in “Proteasome” and “PPAR signaling” pathways, respectively.

## Discussion

HCM is a recognized genetic form of heart disease. Many patients have poor long-term outcomes, including heart failure, malignant arrhythmias and sudden cardiac death. Currently there are no treatments that can effectively reverse the disease, including drug therapy, interventional therapy, and surgical treatment. Therefore, there is an urgent need to explore new effective therapeutic strategies and etiological explanations for HCM. Genetic testing is an indispensable part of labor practice, which has diagnostic and predictive value. It also offers hope that scientists will be able to decipher the mechanisms of disease occurrence and identify targets for effective treatments. As well, new sequencing techniques have led to a large number of candidate genes. In this study, we used a global approach to construct a gene co-expression network to predict candidate gene clusters in the pathogenesis of HCM. Furthermore, we hope to predict candidate gene sets as the basis for a given biological process through closed co-expressed gene modules with common functional annotations.

WGCNA is a powerful statistical method based on gene correlation which can be used to construct gene networks, detect modules, identify central genes and screen candidate genes as biomarkers [16]. In the statistical process, WGCNA focuses on processing a set of gene modules rather than individual genes. This avoids the disadvantages of only processing genes and therefore ignoring molecular transcription networks. A few similar bioinformatic studies have been previously reported. Jing et al. identified specific modules and hub genes related to coronary artery disease by WGCNA (Jing [17]). In 2019, Ran et al. identified biomarkers correlated with hypertrophic cardiomyopathy with co-expression analysis (Ran [4]). In order to avoid the failures of choosing soft thresholding power by scale-free topology fit, we did not filter genes by differential expression when using WGCNA as in previous similar studies. In this study, we obtained 4000 genes of 105 HCM samples from a NCBI dataset, which yielded 7 modules through the use of the WGCNA method. According to a correlation study by the topological overlap matrix (TOM) plot (Fig. 4), each module was shown to be independent of the others. In addition, functional enrichment analysis was performed on the genes in these modules to identify important modules and the genes they contained. By analyzing the functional richness of these seven modules, there are significant differences in their enrichment degrees and terms. By analyzing these data, we found that in both GO enrichment and KEGG pathway analysis, the turquoise module has the highest enrichment. The greatest number of genes (1202 genes) were enriched in the turquoise module. It accounts for 30% of the total number of genes. Therefore, the turquoise module is the most relevant module from the 7 previously identified. Through GO analysis, the genes were mainly concentrated in protein binding, poly(A) RNA binding and translation. Through KEGG analysis, we can find that differentially expressed genes in the turquoise module are highly rich in Ribosome, Proteasome and Oxidative phosphorylation. The WGCNA package function “chooseTopHubInEachModule” was used to identify the modular hub genes with the highest connectivity. The hub genes of each module are RPL35A for module Black, FH for module Blue, PREI3 for module Brown, CREB1 for module Green, LOC641848 for module Pink, MYH7 for module Turquoise and MYL6 for module Yellow. Interestingly, the hub gene of the most important module Turquoise obtained by GO and KEGG analysis was MYH7. It has been repeatedly reported as one of the prevalent pathogenic mutation genes for HCM [1, 22]. Compared to normal people, the above 7 hub genes were further differentially expressed. We found that FH and MYH7 were highly expressed in the HCM group compared to the normal group. These results suggested that these two genes may play an important role in the occurrence and development of HCM. Therefore MYH7 (Fig. 7a) and FH (Fig. 7b) were regarded as true hub genes. Moreover, ROC curves revealed their high diagnostic value as biomarkers for HCM (Fig. 7c; MYH7 AUC: 0.762, FH AUC: 0.612).

MYH7 was a gene encoding myosin heavy chain beta (MHC-β). It was mainly expressed in the heart and is also expressed in skeletal muscles [20]. Multiple previous independent studies have demonstrated that pathogenic mutations in the β-myosin heavy chain (MYH7) gene caused HCM [9, 25]. In addition, mutations in the MYH7 gene are very common in HCM, and can be seen in 25 to 40% of patients [26]. The hub genes in the important modules calculated by WGCNA in this study are consistent with the results of many of the above studies.

The fumarate hydratase (FH) gene is localized to the chromosomal position 1q42.3-q43. In normal cells, the FH gene is located in both mitochondria and cytosol and catalyzes fumarate to malate [14]. Fumarate is a covalent oncometabolite. Its accumulation is characteristic of hereditary leiomyomatosis of genetic cancer syndrome. The mutation of the FH gene may cause the affected cells to transition to aerobic glycolysis (Warburg effect) [15]. It has been found that mutations in fumaric acid can cause several fumarase-related diseases in humans, such as benign mesenchymal tumors of the uterus, leiomyomatosis and so on. In the results of this study, the FH gene may also be a key gene in the occurrence and development of HCM. At present there is little research on their correlation. In the future, exploration of the FH gene may be a salient new direction for further research on HCM.

Therefore, in order to determine the potential molecular function of these two important hub genes, we continued to use GSEA to search for KEGG pathways that are rich in high-expression samples. As shown in Fig. 8a and b, genes in high expression groups of MYH7 and FH were enriched (*p* < 0.05) in “Proteasome” and “PPAR signaling” pathways, respectively.

The proteasome is the main multicatalytic protease complex. It is involved in the degradation of most intracellular proteins. In addition, muscle fibers and sarcoma proteins have been shown to be primarily degraded by proteasome. Some scholars have pointed out that proteasome dysfunction is related to human HCM [19]. As well, a previous study suggested that the protease inhibitor ps-519 may cause a significant regression of cardiac hypertrophy.

Peroxisome proliferator-activated receptors (PPAR) are expressed in many tissues, such as skeletal and cardiac muscles, fat cells, liver cells, etc. Different subtypes of PPAR have different tissue distribution and expression profiles, leading to different clinical outcomes. In particular, the subtype of PPARα is highly expressed in tissues with high fatty acid oxidation capacity, such as liver, heart, and skeletal muscle. In addition, activation of the receptor for another subtype, PPARβ/δ has been shown to protect the myocardium from ischemia-reperfusion injury typical of diabetic cardiomyopathy. Although there is no research on the direct relationship between the PPAR pathway and HCM, this pathway may also be a direction for further researching HCM and finding therapeutic approaches.

## Conclusions

In this study, two key genes of HCM, FH and MYH7, were identified from extensive genetic data through co-expression network analysis. In addition, the most enriched pathways for two key genes were discovered. They are the PPAR signaling pathway and proteasome. They may have played a very important role in the occurrence and development of HCM. The key genes and pathways identified in this study may provide guidance for further study of the mechanism and treatment of HCM in the future. These findings still need to be verified in a large number of clinical practices in the future.

## Data Availability

The datasets generated during the current study are available in the GEO database (http://www.ncbi.nlm.nih.gov/geo).

## Abbreviations

HCM: Hypertrophic cardiomyopathy
WGCNA: Weighted gene co-expression network analysis
GO: Gene ontology
ROC: Receiver operating characteristic
GSEA: Gene set enrichment analysis
TOM: Topological overlap matrix
MHC-β: myosin heavy chain beta
FH: Fumarate hydratase
PPAR: Peroxisome proliferator-activated receptors
